# Anion channelrhodopsins for inhibitory cardiac optogenetics

**DOI:** 10.1038/srep33530

**Published:** 2016-09-15

**Authors:** Elena G. Govorunova, Shane R. Cunha, Oleg A. Sineshchekov, John L. Spudich

**Affiliations:** 1 Center for Membrane Biology, Department of Biochemistry & Molecular Biology, University of Texas Health Science Center at Houston – McGovern Medical School, Houston, Texas, USA; 2 Department of Integrative Biology & Pharmacology, University of Texas Health Science Center at Houston – McGovern Medical School, Houston, Texas, USA

## Abstract

Optical control of the heart muscle is a promising strategy for cardiology because it is more specific than traditional electrical stimulation, and allows a higher temporal resolution than pharmacological interventions. Anion channelrhodopsins (ACRs) from cryptophyte algae expressed in cultured neonatal rat ventricular cardiomyocytes produced inhibitory currents at less than one-thousandth of the light intensity required by previously available optogenetic tools, such as the proton pump archaerhodopsin-3 (Arch). Because of their greater photocurrents, ACRs permitted complete inhibition of cardiomyocyte electrical activity under conditions in which Arch was inefficient. Most importantly, ACR expression allowed precisely controlled shortening of the action potential duration by switching on the light during its repolarization phase, which was not possible with previously used optogenetic tools. Optical shortening of cardiac action potentials may benefit pathophysiology research and the development of optogenetic treatments for cardiac disorders such as the long QT syndrome.

Manipulation of the membrane potential by light using genetically encoded microbial rhodopsins (optogenetics) enables control of defined cell populations^1^,^2^. Therefore optogenetic gene therapy could provide a more targeted and less invasive alternative for cardiomodulation than implanted electrical devices or pharmaceuticals^3^,^4^,^5^. Whereas cardiac pacing by light depends on membrane-depolarizing, excitatory optogenetics tools^6^,^7^,^8^, in cardiology there is also a need for hyperpolarizing, inhibitory molecules. Transgenically expressed halorhodopsin (*Np*HR) was used to identify pacemaker regions in the zebrafish heart^9^. However, *Np*HR and other rhodopsin ion pumps transport only one charge per captured photon, which makes them relatively inefficient silencing tools^10^. Recently channelrhodopsins with strict anion selectivity and high unitary conductance (anion channelrhodopsins, or ACRs) have been discovered in cryptophyte algae and used for neuronal inhibition at much lower light intensities than rhodopsin ion pumps and Cl^−^-conducting mutants of cation channelrhodopsins^11^.

Here we test the performance of ACRs from *Guillardia theta (Gt*ACRs) as tools for inhibition of cardiomyocyte electrical activity and compare them side by side with the proton pump archaerhodopsin-3 (Arch)^12^, previously used for this purpose^13^. Our choice of this pump for comparison with *Gt*ACRs was motivated by the observation that Arch generated larger hyperpolarizing photocurrents than *Np*HR when measured in the same heterologous system^12^,^14^. We show that *Gt*ACRs generated larger photocurrents than Arch when expressed in cultured cardiomyocytes. Complete inhibition of electrical activity was observed in *Gt*ACR-expressing cardiomyocytes at a light intensity which resulted in only a small decrease in the frequency of spiking in Arch-expressing cells. Moreover, by threshold-based closed-loop optogenetics in cardiomyocytes expressing *Gt*ACRs we could accelerate repolarization and precisely terminate action potentials at any time during their repolarization phase, which, to the best of our knowledge, has not been previously demonstrated.

## Results

**Figure 1:**
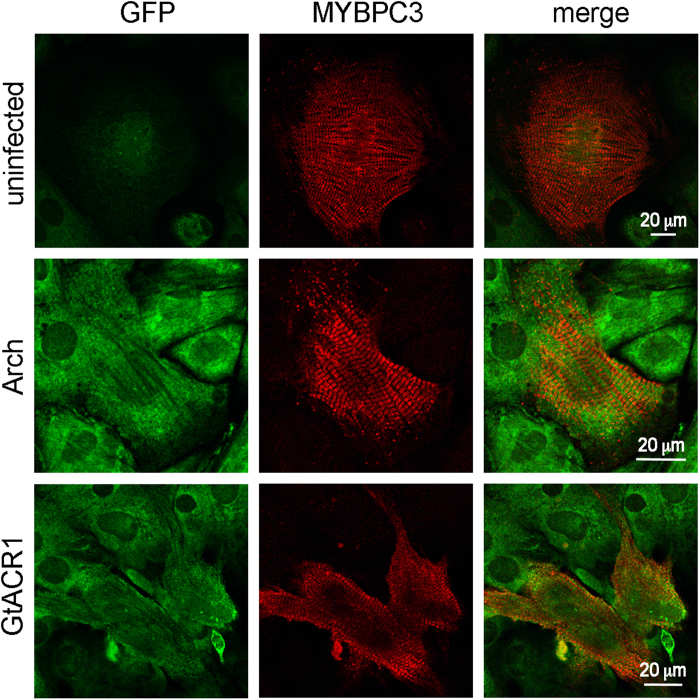
Expression of Arch or *Gt*ACR1 in cultured NRVMs. Confocal immunofluorescence images of uninfected NRVMs (top row), transduced with Arch (middle row) and transduced with *Gt*ACR1 (bottom row). EYFP tag fluorescence is shown in the left column (green channel), the cardiomyocyte marker (MYBPC3, myosin binding protein C-3) fluorescence is shown in the middle column (red channel), and merged images are shown in the right column.

We expressed the transmembrane domains of *Gt*ACR1, *Gt*ACR2 and Arch fused to C-terminal enhanced yellow fluorescence protein (EYFP) under the mouse ubiquitin C promoter in cultured neonatal rat ventricular cardiomyocytes (NRVMs) using lentiviral delivery. The position of the maximal absorption wavelength of *Gt*ACR1 (515 nm) is red-shifted compared to that of *Gt*ACR2 (470 nm), which is advantageous because it allows using light stimuli of higher penetration depth. Therefore, we chose *Gt*ACR1 for comparison with Arch. Expression of both constructs in cardiomyocytes was confirmed by immunofluorescent microscopy (Fig. 1).

**Figure 2:**
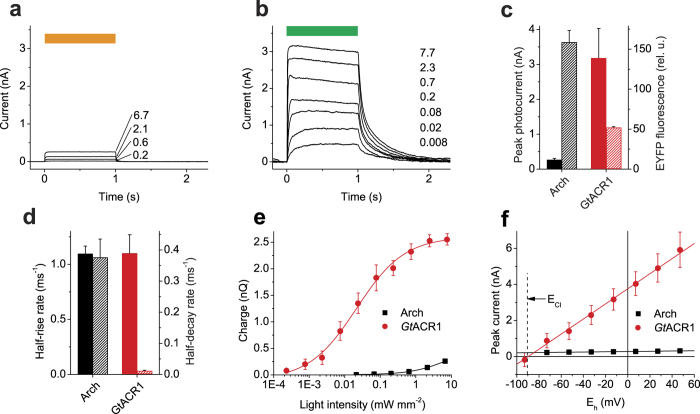
Photocurrents generated by Arch and *Gt*ACR1 in cultured NRVMs. (**a**,**b**) Series of photocurrents generated by Arch (**a**) and *Gt*ACR1 (**b**) at 0 mV voltage at the amplifier output in response to 1-s light pulses shown as the colored bars on top. The wavelength was 560 nm for Arch and 510 nm *Gt*ACR1; the numbers show the intensity in mW mm^−2^. (**c**) The peak amplitude of photocurrents at 0 mV at the amplifier output at 6.7 mW mm^−2^ light intensity (solid bars, left axis) and relative tag fluorescence normalized for the membrane area (hatched bars, right axis). Error bars, sem (*n* = 5 cells). (**d**) The half-rise (solid bars, left axis) and half-decay rates of photocurrents (hatched bars, right axis). Error bars, sem (*n* = 5 cells). (**e**) The dependence of the amount of charge transported across the membrane during 1-s illumination on the stimulus intensity. Error bars, sem (*n* = 5 cells). (**f**) The current-voltage dependence under standard conditions (bath: [Cl^−^] 135 mM, pH 7.3; pipette: [Cl^−^] 4 mM, pH 7.2; other components see in Methods). Error bars, sem (*n* = 3 cells).

First, we characterized photocurrents generated by Arch and *Gt*ACR1 by whole-cell voltage clamp of individual NRVMs. Figure 2a,b show series of hyperpolarizing photocurrents recorded from cells expressing Arch and *Gt*ACR1, respectively, evoked by a 1-s light pulse of varied intensity. The mean peak amplitude of *Gt*ACR1 photocurrents was ~12 fold greater than that of Arch currents, although the expression level of the former, assessed by measuring the tag fluorescence, was ~3 fold lower (Fig. 2c). The current rise and decay rates are shown in Fig. 2d. The membrane hyperpolarization is proportional to the amount of charge transported across the membrane. Figure 2e shows the dependence of charge transported during 1 s of illumination on the light intensity measured in cells expressing Arch or *Gt*ACR1. Almost 4 orders of magnitude less light was needed to reach the same value for *Gt*ACR1 than for Arch. The amplitude of Arch currents showed a weak voltage dependence, whereas that of *Gt*ACR1 currents was very steep (Fig. 2f), making the latter protein a dynamic hyperpolarizing tool at membrane potentials above the Nernst equilibrium potential for Cl^−^.

**Figure 3:**
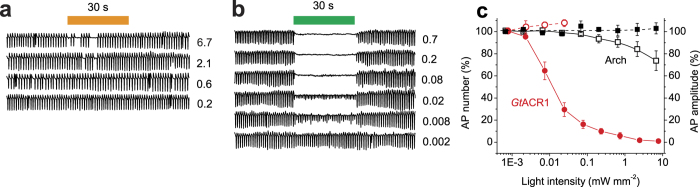
Photoinhibition of electrical activity of cultured NRVMs transduced with Arch or *Gt*ACR1. (**a**,**b**) Extracellular voltage recording from cell clusters expressing Arch (**a**) or *Gt*ACR1 (**b**) normalized to the mean value of the AP amplitude recorded before the onset of illumination. The 30-s light pulse is schematically shown as the colored bar on top. The numbers show the light intensity in mW mm^−2^. (**c**) The light dependence of AP number during the illumination period relative to the previous dark period (left axis, empty symbols) and AP amplitude (right axis, filled symbols) cells expressing Arch (black) and *Gt*ACR1 (red). Error bars, sem (*n* = 5–9 cells).

To test the performance of Arch and *Gt*ACR1 as tools for inhibition of cardiomyocyte function without perturbing the intracellular ion concentrations, we used extracellular recording from star-shaped aggregates of synchronously beating cells in growth medium. Expression of either Arch or *Gt*ACR1 did not change the frequency of APs in the dark, which was 67 ± 3 (mean ± sem; *n* = 11 cell clusters) and 68 ± 3 (mean ± sem; *n* = 29 cell clusters) APs/min, respectively, compared to 65 ± 2 (mean ± sem; *n* = 6 cell clusters) for control (uninfected) NRVMs. In cells expressing Arch switching on the light resulted in immediate suppression of electrical activity, but it was resumed at irregular intervals during a 30-s illumination period even at the highest intensity used (6.7 mW mm^−2^; Fig. 3a). In cells expressing *Gt*ACR1 illumination caused a decrease in AP amplitude; 50% inhibition was observed at ~10 μW mm^−2^ (Fig. 3b). Figure 3c summarizes the results obtained with Arch- and *Gt*ACR1-expressing cells. In control (uninfected) NRVMs both the AP frequency and amplitude were unaffected by light even at the maximal intensity: 96 ± 3% and 108 ± 6%, respectively, as compared to the previous dark period (mean ± sem; *n* = 6 cell clusters). Normal electrical activity of the *Gt*ACR1-expressing culture restored within 2.0 ± 0.4 s at 7.7 mW mm^−2^ (mean ± sem; *n* = 6 cell clusters) and 1.5 ± 0.2 s at 0.7 mW mm^−2^ (mean ± sem; *n* = 5 cell clusters) after switching off the light of the maximal intensity. In Arch-expressing NRVMs no significant increase in AP frequency was observed after switching off the light relative to the preillumination period, in contrast to the results obtained in cardiomyocytes electrically coupled to Arch-expressing human embryonic kidney (HEK293) cells^13^.

**Figure 4:**
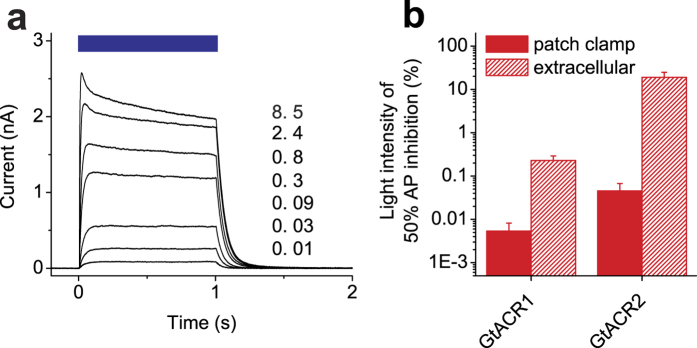
Photocurrents generated by *Gt*ACR2 and comparison of the efficiency of photoinhibition of AP generation by *Gt*ACR1 and *Gt*ACR2. (**a**) A series of photocurrents generated by *Gt*ACR2 at 0 mV voltage at the amplifier output in response to 1-s light pulses shown as the colored bars on top. The wavelength was 470 nm; the numbers show the intensity in mW mm^−2^. (**b**) Comparison of the light intensities required for 50% inhibition of AP amplitude in NRVMs expressing *Gt*ACR1 or *Gt*ACR2 determined by extracellular recording (hatched bars) or whole-cell patch clamp in current clamp mode (solid bars). Error bars, sem (*n* = 3–9 cells).

*Gt*ACR2 also generated robust photocurrents in NRVMs (Fig. 4a), but more light was required to achieve the same degree of AP inhibition as compared to *Gt*ACR1-expressing cells (Fig. 4b, hatched bars). The decay rate of *Gt*ACR2-generated photocurrent at 0 mV was 0.019 ± 0.004 ms^−1^ (*n* = 5 cells), i.e., ~2 fold higher than that of *Gt*ACR1-generated currents (Fig. 2d). We also compared AP inhibition in *Gt*ACR1- and *Gt*ACR2-expressing cells by patch clamp recording in the current clamp mode. For both proteins, the light sensitivity probed by patch clamping was higher than that determined noninvasively by extracellular recording (Fig. 4b, solid bars), which can be explained by a steeper Cl^−^ gradient used in patch clamp experiments. Measurements of the intracellular Cl^−^ concentration in the heart muscle by ion-selective microelectrodes yielded values from 14 to 20 mM depending on the animal species, cell type and developmental stage^15^,^16^,^17^. We tested the dependence of the photocurrent amplitude and the reversal potential (E_rev_) on the Cl^−^ concentration in the pipette. When it was increased from 4 to 40 mM, the E_rev_ followed the Nernst equilibrium potential for Cl^−^, as expected, which resulted in ~2-fold reduction of the photocurrents (Supplementary Fig. S1).

**Figure 5:**
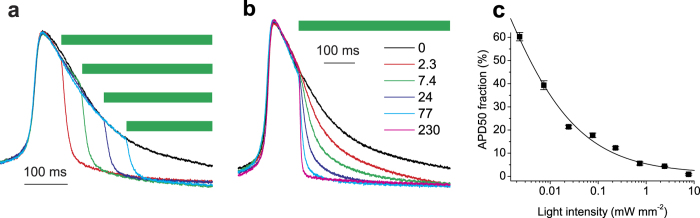
Optical shortening of APs in cultured NRVMs expressing *Gt*ACR1. (**a**,**b**) Normalized APs recorded from NRVMs expressing *Gt*ACR1 in the dark (black lines) and upon illumination (colored lines) with 510-nm light shown as green bars. In (**a**) the light pulse (77 μW mm^−2^) was delivered at different times during the repolarization phase of the AP. In (**b**) the light pulses of varied intensity (numbers in the legend, in μW mm^−2^) were delivered at the same time point. (**c**) Ensemble data from experiments as in panel (**b**). The APD50 fraction from the onset of the light was measured and divided by the value measured from the same time point in the dark. Error bars, sem (*n* = 5 APs). The line is a Lorentz function approximation.

Besides complete silencing of cardiac electrical activity, optogenetics can potentially be used for shaping AP morphology^18^. In particular, changing the AP duration (APD) is desired for probing and potentially curing pathological conditions resulting from APD abnormalities, such as the life-threatening long QT syndrome, which results from an abnormally slow repolarization phase of the AP^19^. To explore the possibility of optical shortening of the APD, we delivered light pulses at different times during an ongoing spontaneous AP using threshold-based closed-loop control. Expression of *Gt*ACRs did not significantly influence the APD at 50% repolarization (APD50) measured in the dark (272 ± 34, 270 ± 32 and 269 ± 21 ms in control, *Gt*ACR1- and *Gt*ACR2-expressing cells, respectively; *n* = 5–7 cells). When light was switched on during the depolarization phase, it resulted in cutting off the nascent AP (Supplementary Fig. S2), as was previously observed using *Np*HR^20^. Moreover, *Gt*ACRs permitted temporally precise truncation of the AP at any time point during the repolarization phase (Fig. 5a and Supplementary Fig. S2), which could not be achieved with *Np*HR^20^. Variation of the light intensity allowed fine tuning of the AP shape (Fig. 5b,c). The AP amplitude in experiments in which the light was switched on during the repolarization phase was 100 ± 0.8% (mean ± sem, *n* = 13 recordings) as compared to that measured in the dark.

## Discussion

In previous studies the proton-pumping rhodopsin Arch was used to suppress cardiomyocyte electrical activity via cell delivery, i.e. by coupling of Arch-expressing HEK293 cells to cardiomyocytes^13^. We showed that AP generation could be suppressed immediately after switching on the light upon lentiviral expression of Arch in cardiomyocytes, but it resumed at irregular intervals during the 30-s illumination period even at the highest light intensity used (6.7 mW mm^−2^). Expression of the chloride-pumping rhodopsin *Np*HR enabled inhibition of individual cardiac APs by short light pulses of lower intensity, but the activity was not monitored for longer illumination periods^20^. For a complete suppression of AP generation during several seconds with *Np*HR a light intensity of 30 mW mm^−2^ was required^9^.

*Gt*ACRs generated much larger currents and permitted AP inhibition at much lower light intensities than Arch. In addition to conducting many ions per photocycle in contrast to only one transported by rhodopsin ion pumps such as Arch or *Np*HR, ACRs also become more efficient upon the membrane depolarization (e.g. during the depolarization phase of an AP) because of the steep voltage dependence of their currents. Unlike rhodopsin ion pumps, ACRs transport ions passively, i.e., they generate hyperpolarizing currents only above the Nernst equilibrium potential for Cl^−^. This means that their efficiency as optogenetic silencing tools will depend on the intracellular Cl^−^ concentration which may vary between species, cell types and other conditions. Nevertheless, currents generated by *Gt*ACR1 at 40 mM Cl^−^ in the pipette (which is twice as high as the intracellular Cl- concentration measured in the heart with ion-selective microelectrodes^15^) were still ~5 fold larger than maximal Arch photocurrents. Furthermore, our data obtained by extracellular recording unambiguously demonstrated that complete inhibition of cardiomyocyte electrical activity by light could be achieved at physiological intracellular Cl^−^ concentrations.

The potentially most attractive application of *Gt*ACRs in optogenetics is their use for temporally precise truncation of APs during the repolarization phase. In a previous study *Np*HR failed to deliver this benefit^20^. Moreover, shortening of the AP duration observed when the light pulse was delivered during the depolarization phase was accompanied by a reduction of the AP amplitude^20^. Therefore, the results obtained with *Np*HR are better described as AP inhibition rather than AP shaping.

Shortening of the AP duration to treat the long QT syndrome appears to be a therapeutic therapeutic goal for which ACRs are particularly suitable. Using intensity-modulated rather than continuous light stimuli may help recreate more realistic morphology of shortened APs. Although continuous illumination of ACR-expressing cardiomyocytes led to a decrease in the AP amplitude rather than frequency (Fig. 3c), potentially closed-loop optogenetics can also be used to reduce the frequency by imposing a desired refractory period after each AP, which can lead to the development of novel therapeutic treatments for tachyarrhythmias.

A disadvantage of *Gt*ACRs as compared to rhodopsin ion pumps is a slower decay rate of their photocurrents. It is of little importance when a complete inhibition of cardiac electrical activity for an extended period of time is required (i.e., for analysis of the heart conduction system as in ref. 9). *Gt*ACR2 exhibits ~2 fold faster photocurrent decay compared to *Gt*ACR1 and is therefore more suitable for the latter goal. Screening of natural ACR variants from other cryptophyte algae and/or molecular engineering of known ACRs is desirable for improving their temporal resolution.

## Methods

### Expression constructs

The polynucleotide sequences encoding the seven-transmembrane (7TM) domains of *Gt*ACR1 (295 amino acid residues), *Gt*ACR2 (291 amino acid residues) and Arch (a gift of E.S. Boyden; 258 amino acid residues) were subcloned to the pFUGW vector backbone^21^ in frame with an enhanced yellow fluorescence protein (EYFP) tag and verified by sequencing.

### Neonatal rat ventricular cardiomyocyte isolation and transduction

Cardiomyocytes were isolated following the procedure as previously described^22^. In accordance with the animal protocol approved by the Animal Welfare Committee at University of Texas Health Science Center at Houston, 1-2 day old Sprague-Dawley rat pups were euthanized by decapitation. Hearts were exposed, excised, minced, and digested through a series of agitations in buffer containing collagenase type II (Worthington, Collagenase type II 305 U/mg) and pancreatin (Sigma). Cells from each digestion were collected and pooled, and pre-plated on Nunc plates (Thermo Fisher Scientific) in Complete Medium (40% DMEM, 40% Ham’s F-10, 20% fetal calf serum) for 2–4 hours to reduce fibroblasts and enrich for cardiomyocytes. Following the pre-plate step, the non-adherent cells (cardiomyocytes) were aspirated from the plate, pelleted, resuspended in Complete Medium, and plated on coverslips or MatTek tissue culture plates (fibronectin-coated; Roche Applied Science). 36 hours later cardiomyocytes were washed with Ham’s F-10 and incubated with lentiviral preparations of Arch, *Gt*ARC1 or *Gt*ARC2 for 12–16 hours. Then, lentivirus was aspirated and the cardiomyocytes were maintained in Complete Medium before experiments were performed. Exogenous all-*trans* retinal (1 μM final concentration) was supplied immediately after transduction to improve light responsiveness of cardiac cells that express channelrhodopsins^23^.

### Confocal immunofluorescence microscopy

48 hours after viral transduction, cardiomyocytes were fixed in 2% paraformaldehyde and incubated with primary antibodies overnight at 4 °C in 1XPBS containing 5% normal goat serum and 0.075% TritonX-100. Primary antibodies used were: MyBPC3 (1:250, Santa Cruz) and GFP (1:500, GF28R, Thermo Scientific). Secondary antibodies used were donkey anti-mouse conjugated to Alexa Fluor 488 and donkey anti-rabbit conjugated to Alexa Fluor 568 (1:500, LifeTechnologies). Images were obtained with a Nikon A1 confocal microscope (Nikon, Melville, NY) equipped with 40x oil, numerical aperture 1.3 objective.

### Fluorescence measurements

EYFP fluorescence was measured with a CoolSnap HQ2 CCD camera (Photometrics, Tucson, AZ) using a Nikon Eclipse Ti-U microscope. Excitation was from an X-Cite 120Q light source (EXFO, Mississauga, Ontario), and fluorescence was detected using a Nikon B-2E/C filter set. All images were taken with the same acquisition parameters and analyzed with the ImageJ1.42q software.

### Patch clamp recording

Patch clamp measurements were performed on NRVMs 48–72 h after transduction using an Axopatch 200B amplifier (Molecular Devices, Union City, CA) at 25 °C. Only cells that spontaneously generated the action potentials (APs) were used for electrophysiological measurements. Such cells constituted ~90% in the culture, which confirmed the efficiency of the cardiomyocyte enrichment protocol. The signals were digitized with a Digidata 1440A using pClamp 10 software (both from Molecular Devices). Patch pipettes with resistances of 2–5 MΩ were fabricated from borosilicate glass. The Tyrode’s bath solution contained (in mM): NaCl 126, KCl 2, MgCl_2_ 1, CaCl_2_ 3, glucose 30, HEPES 25 (pH 7.3 adjusted with NaOH). The pipette solution contained, unless otherwise indicated, (in mM): K gluconate 135, MgCl_2_ 2, HEPES 20 (pH 7.2). A 4 M salt bridge was used in all experiments. The current-voltages dependencies were corrected for liquid junction potentials calculated using the ClampEx built-in LJP calculator. Continuous light pulses were provided by a Polychrome IV light source (T.I.L.L. Photonics GMBH, Grafelfing, Germany) in combination with a mechanical shutter (Uniblitz Model LS6, Vincent Associates, Rochester, NY; half-opening time 0.5 ms). The light intensity was attenuated with the built-in Polychrome system or with neutral density filters. Maximal quantum density at the focal plane of the 40x objective lens was 7.7 mW/mm^2^ for 510-nm light and 6.7 mW/mm^2^ for 560-nm light. Threshold-based closed-loop control was implemented by triggering a 5-V pulse at ~95% of the AP peak to open the shutter after a time delay set by an S44 electrical stimulator (Grass Medical Instruments, Quincy, MA). Data were analyzed using pClamp 10 and Origin 7 (OriginLab Corporation, Northampton, MA) software.

### Extracellular recording

Extracellular electrical recording was performed on clusters of synchronously beating NRVMs in the growth medium at 37 °C (controlled by an automatic temperature controller TC-324B, Warner Instruments Corporation, Hamden, CT) 7–10 days after transduction using the same equipment as described for patch clamping. The pipette was filled with the Tyrode’s solution and placed in the vicinity of a cell cluster so that it did not perturb its contraction. The electrical activity was recorded for 30 s in the dark to assess the baseline, after which a 30-s light pulse was applied followed by another 30-s dark period to monitor the recovery of beating after illumination. For analysis, the low-pass filter at 0.3 Hz and a high-pass filter at 3 Hz were applied to the recorded traces using pClamp 10.

### Statistics

Statistical data are shown as mean ± sem. The n values in the legends indicate the number of independent experiments (individual cardiomyocytes or their clusters). For analysis of significance unpaired Student’s t-test was used, and p values less than 0.05 were considered significant.

## Additional Information

**How to cite this article**: Govorunova, E. G. *et al.* Anion channelrhodopsins for inhibitory cardiac optogenetics. *Sci. Rep.* **6**, 33530; doi: 10.1038/srep33530 (2016).

## Supplementary Material

Supplementary Information
