# Supplementary material for: Anion channelrhodopsins for inhibitory cardiac optogenetics
Source: Sci Rep. 2016 Sep 15;6:33530. doi: 10.1038/srep33530 (PMC5024162; doi:10.1038/srep33530)
Supplement: Supplementary Information [file srep33530-s1.pdf]

Supplementary Material

Anion channelrhodopsins for inhibitory cardiac optogenetics

Elena G. Govorunova[1], Shane R. Cunha[2], Oleg A. Sineshchekov[1] & John L. Spudich[1*]

[1]Center for Membrane Biology, Department of Biochemistry & Molecular Biology, University of Texas Health Science Center at Houston – McGovern Medical School, Houston, Texas, USA; [2]Department of Integrative Biology & Pharmacology, University of Texas Health Science Center at Houston – McGovern Medical School, Houston, Texas, USA.

[*]Correspondence should be addressed to J.L.S. (John.L.Spudich@uth.tmc.edu).

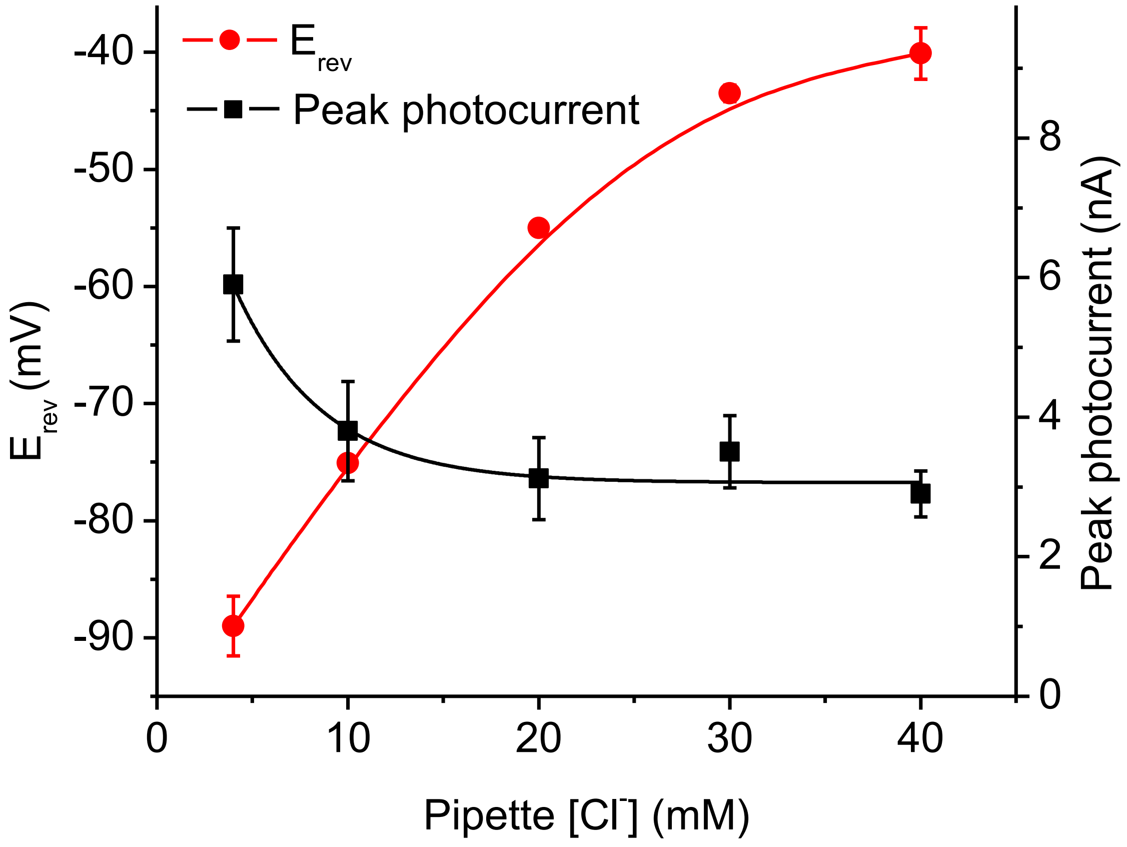

Supplementary Figure S1. The dependence of the reversal potential, E_{rev} (left axis) and the mean peak photocurrent at 0 mV (right axis) generated by *Gt*ACR1 in NRVMs on the Cl^- concentration in the pipette. The data points are the mean values ± sem (*n* = 3-4 cells for E_{rev} and 6-8 cells for peak currents).

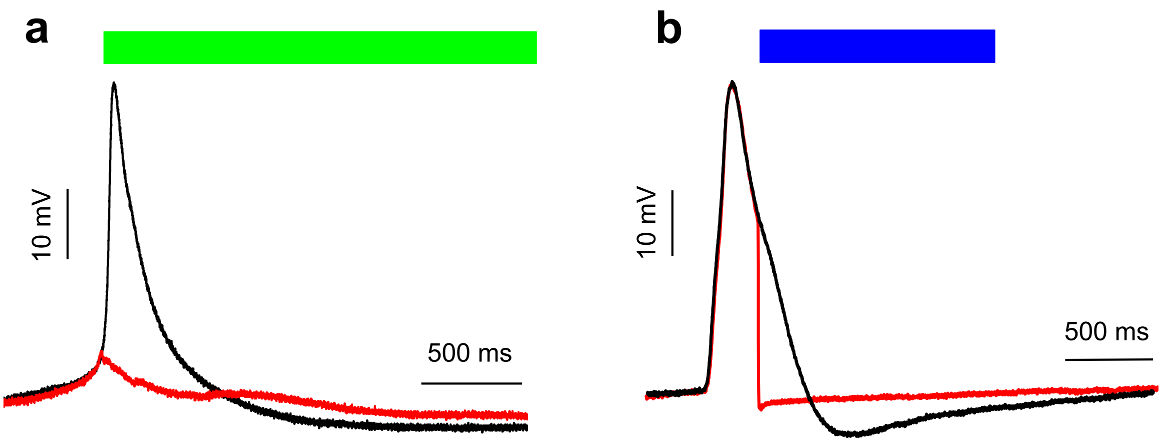

Supplementary Figure S2. The influence of light on the AP shape in NRVMs expressing *Gt*ACRs. (a) Inhibition of an AP by switching on the light during the depolarization phase in a *Gt*ACR1-expressing cell. Light (schematically shown as the green bar): 510 nm, 230 µW mm^{-2}. (b) Shortening of the AP duration by switching on the light during the repolarization phase in a *Gt*ACR2-expressing cell. Light (schematically shown as the blue bar): 470 nm, 250 µW mm^{-2}. In both panels the red lines show current traces recorded upon illumination, the black lines, traces recorded from the same cell in the dark.
